# GPR84 aggravates lung inflammation through activating ZBP1-PANoptosome mediated PANoptosis following IAV infection

**DOI:** 10.1038/s41420-026-03158-z

**Published:** 2026-06-12

**Authors:** Hui Jiang, Youqin Zeng, Shijun Xu, Pengfei Wang, Shengjie You, Xiaobo Zhang, Hao Zhou

**Affiliations:** 1 School of Basic Medicine, Chengdu University of Traditional Chinese Medicine, Chengdu, China; 2 College of Medical Technology, Chengdu University of Traditional Chinese Medicine, Chengdu, China; 3 School of Pharmacy, Chengdu University of Traditional Chinese Medicine, Chengdu, China; 4 School of Life Sciences, Fudan University, Shanghai, China; 5 Chongqing Taiji Industry (Group) Co., Ltd., Chongqing, China

**Keywords:** Immunology, Apoptosis, Cell signalling

## Abstract

Influenza virus-induced pneumonia (IVP) is a contagious lung disease marked by severe lung inflammation following viral infection and remains a significant public health concern due to its high mortality rate. G protein-coupled receptor 84 (GPR84) has been implicated in various inflammatory diseases, however, its role in influenza virus-induced lung inflammation remains poorly understood. In this study, using high-throughput screening, we found that Influenza A virus (IAV) infection markedly upregulates the expression of several G protein-coupled receptors, with GPR84 mRNA and protein levels being highly induced in the lungs of animal models during pneumonia. Mechanistically, GPR84 enhances ZBP1-PANoptosome mediated PANoptosis and exacerbates the release of inflammatory chemokines and danger associated molecular patterns (DAMPs). Notably, deletion of GPR84 attenuates influenza virus-induced PANoptosis, indicating that GPR84 participates in regulating lung inflammation and the pathogenesis of pneumonia during influenza infection. Overall, these findings demonstrate that GPR84 plays a central role in influenza virus–induced lung inflammation by promoting PANoptosis and the release of inflammatory mediators. Targeting GPR84 attenuates IAV-induced PANoptosis, highlighting its potential as a therapeutic target to mitigate the pathogenesis of influenza virus-induced pneumonia.

## Introduction

Influenza A virus (IAV) is responsible for annual seasonal epidemics or global pandemics, resulting in millions of infections and even deaths worldwide [1, 2]. IAV infection leads to the release of large amounts of antiviral and inflammatory cytokines, triggering immune dysregulation. One of the major fatal complications is influenza virus-induced pneumonia (IVP), characterized by excessive lung inflammation due to viral invasion. After being recognized by multiple pattern recognition receptors (PRRs), IAV triggers overwhelming cytokine response, resulting in a “cytokine storm” that disrupts immune homeostasis and contributes to severe pneumonia in infected individuals [3]. Programmed cell death plays a crucial role in host defense and immune regulation. PANoptosis, a recently identified form of regulated cell death, involves simultaneous activation of apoptosis, pyroptosis, and necroptosis [4]. Apoptosis is characterized by its apoptotic morphology and does not elicit an inflammatory response, pyroptosis is marked by cell membrane rupture, release of inflammatory contents, and inflammasomes formation. Necroptosis is characterized by organelle swelling and membranes rupture [5, 6]. Z-DNA-binding protein 1 (ZBP1), known as a viral recognition protein, can interact with receptor-interacting protein kinase 3 (RIPK3) and receptor-interacting serine/threonine-protein kinase 1 (RIPK1) to trigger PANoptosis [7, 8]. ZBP1 activates components such as NLRP3 inflammasome, mixed lineage kinase domain-like protein (MLKL), and caspase-8 to promote pyroptosis, necroptosis, and apoptosis, respectively [9, 10]. During IAV infection, ZBP1 forms the PANoptosome, initiating PANoptosis in response to viral detection [11–13]. Notably, inhibition of PANoptosis has been shown to alleviate lung inflammation [14]. However, the presence and therapeutic targeting of PANoptosis in IAV-induced inflammation remain insufficiently understood.

To investigate how IAV initiates PANoptotic signaling pathways during IAV infection, we performed an initial high-throutput screening analysis of lung tissues collected from H1N1 infected models. As expected, we observed a pronounced cytokine storm. Notably, we also identified significant dysregulation of G protein-coupled receptors (GPRs), a large family of therapeutic targets implicated in diverse pathological conditions, such as diabetic skin wounds [15], hepatic fibrosis [16], colorectal cancer [17]. GPRs are key regulators of physiological and pathological processes and are widely recognized as drug targets, including GPR40 (FFAR1), GPR41 (FFAR3), GPR43 (FFAR2), GPR84, and GPR120 (FFAR4) [18]. Among them, G protein coupled receptor 84 (GPR84) is a pro-inflammatory receptor highly expressed on immune system cells such as neutrophils, macrophages, and its expression is upregulated in response to inflammatory stimuli [18–23]. GPR84 acts as an amplifier of inflammatory signaling by enhancing the release of inflammatory mediators (such as TNF-α and various interleukins) [20]. GPR84 was found to be involved in inflammatory conditions, including gastroesophageal reflux disease [24], inflammatory bowel disease [25]. and neuropathic pain [26]. In addition, preliminary evidence links GPR84 to pathological outcomes such as organ fibrosis, leukemia, as well as osteopoiesis [27–29]. Upon stimulation with lipopolysaccharide (LPS) or TNF-α, GPR84 expression increases markedly [30]. In contrast, GPR84 deletion in macrophages reduced production of IL-1β, IL-6, and TNF-α after LPS stimulation [19, 21, 31], and lead to increased Th2 cytokines in mice CD4^+^ T lymphocytes [32]. In recent years, the role of GPR84 in viral infection and the mechanisms by which it precisely regulates inflammation in these settings have remained largely uncharacterized. Notably, a rigorous evaluation of whether GPR84 signaling would affect PANoptosis-mediated inflammatory responses during viral infection highlights a knowledge gap regarding its biological function and druggable potentials in both cellular and preclinical animal studies.

Despite these findings described above, the precise mechanism through which GPR84 regulates PANoptosis-driven inflammatory responses during influenza viral pneumonia remains unclear. In this study, we observed that viral infection induces excessive secretion of inflammatory cytokines, chemokines, and damage-associated molecular patterns (DAMPs), including HMGB1, S100A8, and S100A9, through activation of ZBP1-mediated PANoptosis. In addition, we found that GPR84 expression is significantly elevated following IAV infection, where it facilitates PANoptosis and aggravates lung inflammation. These findings further broaden our understanding of the role of GPR84 in IAV-induced pneumonia during H1N1 infection.

## Results

### H1N1 infection causes lung damage and inflammation

**Fig. 1:**
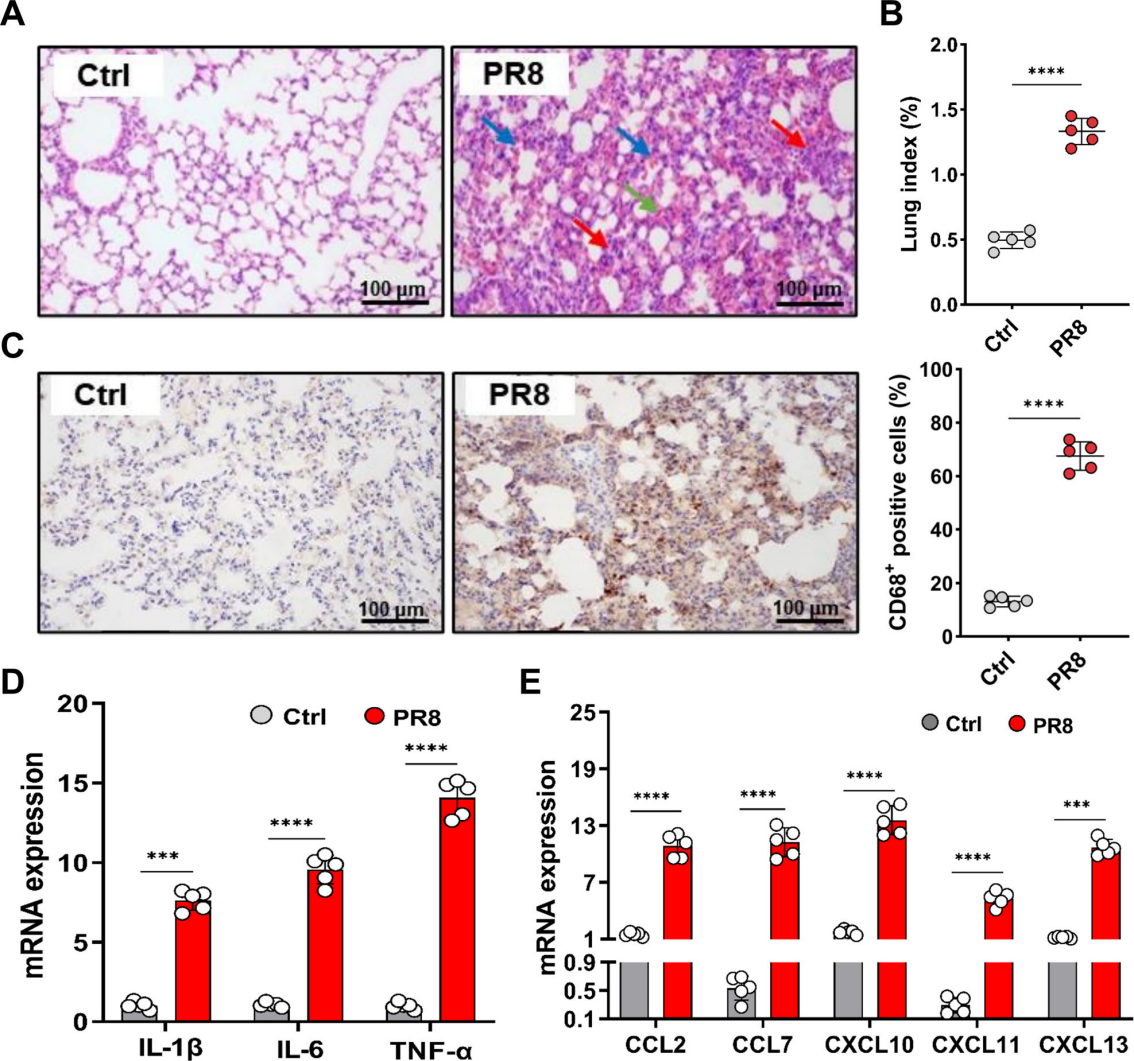
H1N1 PR8 infection induces lung tissue damage. Six-week-old female BALB/c mice were inoculated with 10 LD_50_ PR8 virus intranasally. One group of mice administered PBS were mock-infected as control, and the other group of mice were infected with PR8 virus. **A** Pathological morphology analysis of representative lung tissues on 5 dpi by H&E staining (*n* = 3 per group). The red arrows indicate neutrophil infiltration, the blue arrows indicate extravasated red blood cells and the green arrows indicate lymphocyte infiltration. Scale bar = 100 μm. **B** Lung tissue was collected on 5 dpi and the lung wet index was calculated as lung wet weight / body weight (*n* = 5). **C** Immunohistochemical detection of CD68 in lung tissues at 5 dpi. Representative images are shown (scale bar = 100 μm), and the number of CD68^+^ positive cells was quantified (right panel; *n* = 5 tissues per group). **D** mRNA expression levels of IL-1β, IL-6, and TNF-α in lung homogenates at 5 dpi (*n* = 5). **E** mRNA expression levels of CCL2, CCL7, CXCL10, CXCL11, and CXCL13, genes were detected by qRT-PCR (*n* = 5). The above data shows an average ± SD. *** *p* < 0.001, **** *p* < 0.0001 compared to the control group.

A lethal infection model using 6–8-week-old female BALB/c mice was established to investigate how IAV causes lung damage and inflammatory responses in vivo. Five days post infection (5 dpi), lung inflammation and damage induced by H1N1 PR8 were evaluated by H&E staining. The results revealed that PR8 -infected mice exhibited notable neutrophil infiltration (red arrows), red blood cells extravasation (blue arrows), and lymphocytic infiltration (green arrows) in the bronchi, compared with the control (Fig. 1A). Additionally, the lung index was increased approximately 1.3-fold in PR8-infected mice (Fig. 1B). Immunohistochemistry analysis further demonstrated elevated CD68 expression in lungs of PR8-infected mice compared to uninfected controls (Fig. 1C). Moreover, mRNA levels of inflammatory cytokines IL-1β (8-fold), IL-6 (10-fold), and TNF-α (14-fold) were significantly elevated in infected lungs (Fig. 1D). Similarly, chemokines such as CCL2 (11-fold), CCL7 (12-fold), CXCL10 (14-fold), CXCL11 (6-fold), and CXCL13 (10-fold) were markedly upregulated (Fig. 1E). In addition, the protein levels of pro-inflammatory factors (IL-1β, IL-6, and TNF-α) were significantly increased in lung tissues following IAV infection (Fig. S1B). Consistently, ELISA analysis revealed that IAV infection markedly increased the secretion of these cytokines, as well as chemokines CCL2, CXCL10, and CXCL13, in serum, BALF, and lung tissue (Fig. S1C, D), with TNF-α and CXCL13 exhibiting particularly pronounced increases. These findings indicate that H1N1 infection induces severe lung inflammation characterized by macrophage infiltration and elevated cytokine and chemokine release.

### IAV infection triggers PANoptosis in cell and animal model

**Fig. 2:**
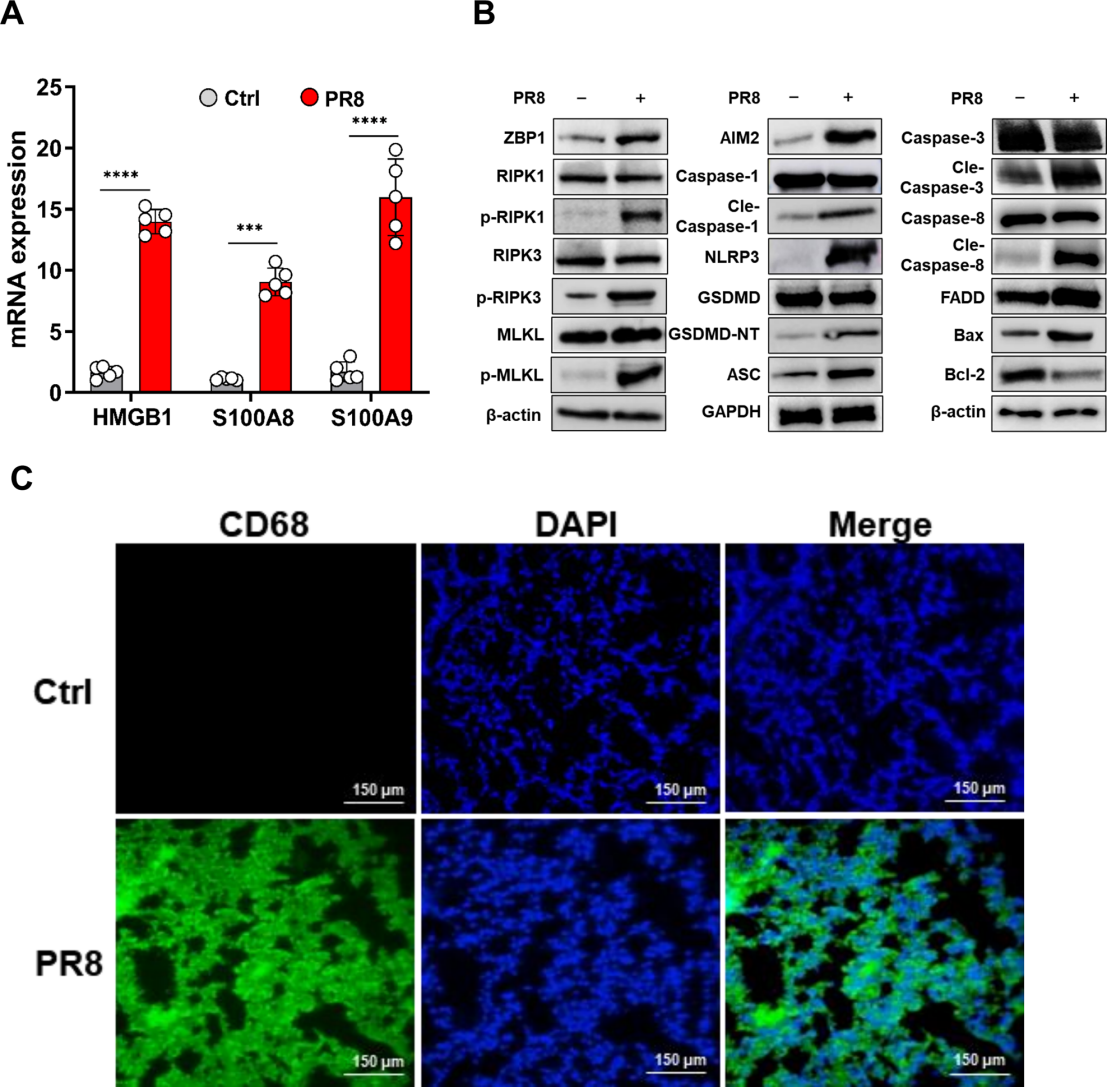
IAV infection leads to apoptosis, necroptosis, and pyroptosis in lung tissue. **A** The mRNA levels of HMGB1, S100A8, and S100A9 in lung homogenates on 5 dpi (*n* = 5). **B** Western blot analysis of PANoptosis- and cell death-associated proteins in lung tissues, including ZBP1, RIPK1, phosphorylated RIPK1 (p-RIPK1), RIPK3, phosphorylated RIPK3 (p-RIPK3), MLKL, phosphorylated MLKL (p-MLKL), AIM2, caspase-1, cleaved caspase-1, NLRP3, GSDMD, GSDMD-NT, ASC, caspase-3, cleaved caspase-3, caspase-8, cleaved caspase-8, and FADD. **C** Immunofluorescence staining of CD68 (green) in lung sections of mice. Nuclei were stained with DAPI (blue). Scale bar = 150 μm. The above data shows an average ± SD. ***p < 0.001, ****p < 0.0001 compared to the control group.

**Fig. 3:**
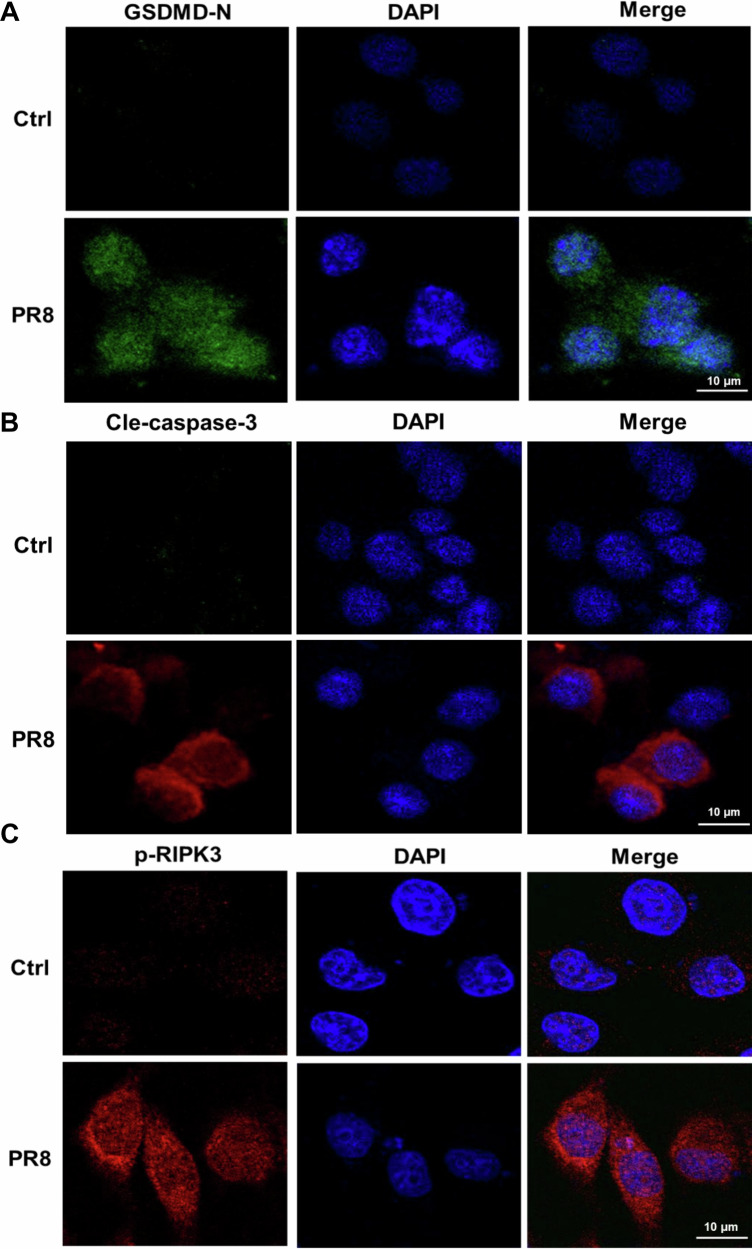
IAV-induced PANoptosis formation. **A**, **B** Immunofluorescence images of the GSDMD-N (green) and cle-caspase-3 (red) in RAW 264.7 cells infected with H1N1 for 12 h. Nuclei (blue) are shown by Hoechst 33342 (scale bar = 10 μm). **C** Confocal microscopy of the p-RIPK3 (red) in A549 cells infected with H1N1 for 12 h. Hoechst 33342 dye (blue) was used to stain the nuclei of cells (scale bar = 10 μm).

Inflammation is often accompanied by excessive release of the DAMPs. We first evaluated the expression of three representative DAMPs, HMGB1, S100A8, and S100A9, and found that their transcription and protein levels were significantly elevated in infected lung tissue, indicating severe damage (Figs. 2A and S1A). PANoptosis, a coordinated cell death program combining pyroptosis, apoptosis, and necroptosis, is a key response to DAMPs and viral infection. To assess whether IAV infection induces PANoptosis, A549 cells were infected with IAV (MOI = 0.5), and levels of PANoptosis markers were measured. IAV infection significantly upregulated ZBP1, a pattern recognition receptor for IAV, along with pyroptosis markers (cleaved caspase-1, NLRP3, GSDMD-NT, ASC), apoptosis markers (cleaved caspase-3, cleaved caspase-8, FADD, Bax and Bcl-2), and necroptosis-related proteins (phosphorylated RIPK1, RIPK3, and MLKL) (Fig. 2B). Immunohistochemical staining for the macrophage marker CD68 indicated a significantly higher macrophage count in infected lungs compared to controls (Fig. 2C). To further investigate how H1N1 infection regulates pyroptosis, apoptosis, and necroptosis in A549, RAW264.7, and lung tissues, immunofluorescence staining with PANoptosis markers, including GSDMD-NT (pyroptosis), cle-caspase-3 (apoptosis), and p-RIPK3 (necroptosis), were performed. The results show that IAV stimulation significantly increased GSDMD and caspase-3 cleavage in RAW264.7, RIPK3 phosphorylation in A549 (Fig. 3A–C). Consistently, our results also show that upregulation of GSDMD-NT, cle-caspase-3, and p-RIPK3 in lung tissue at 5 dpi H1N1 infection (Fig. S2A–C). These data suggest that IAV infection triggers PANoptosis, contributing to inflammatory mediator release.

### ZBP1 mediates IAV-induced PANoptosis and exacerbates chemokine production

**Fig. 4:**
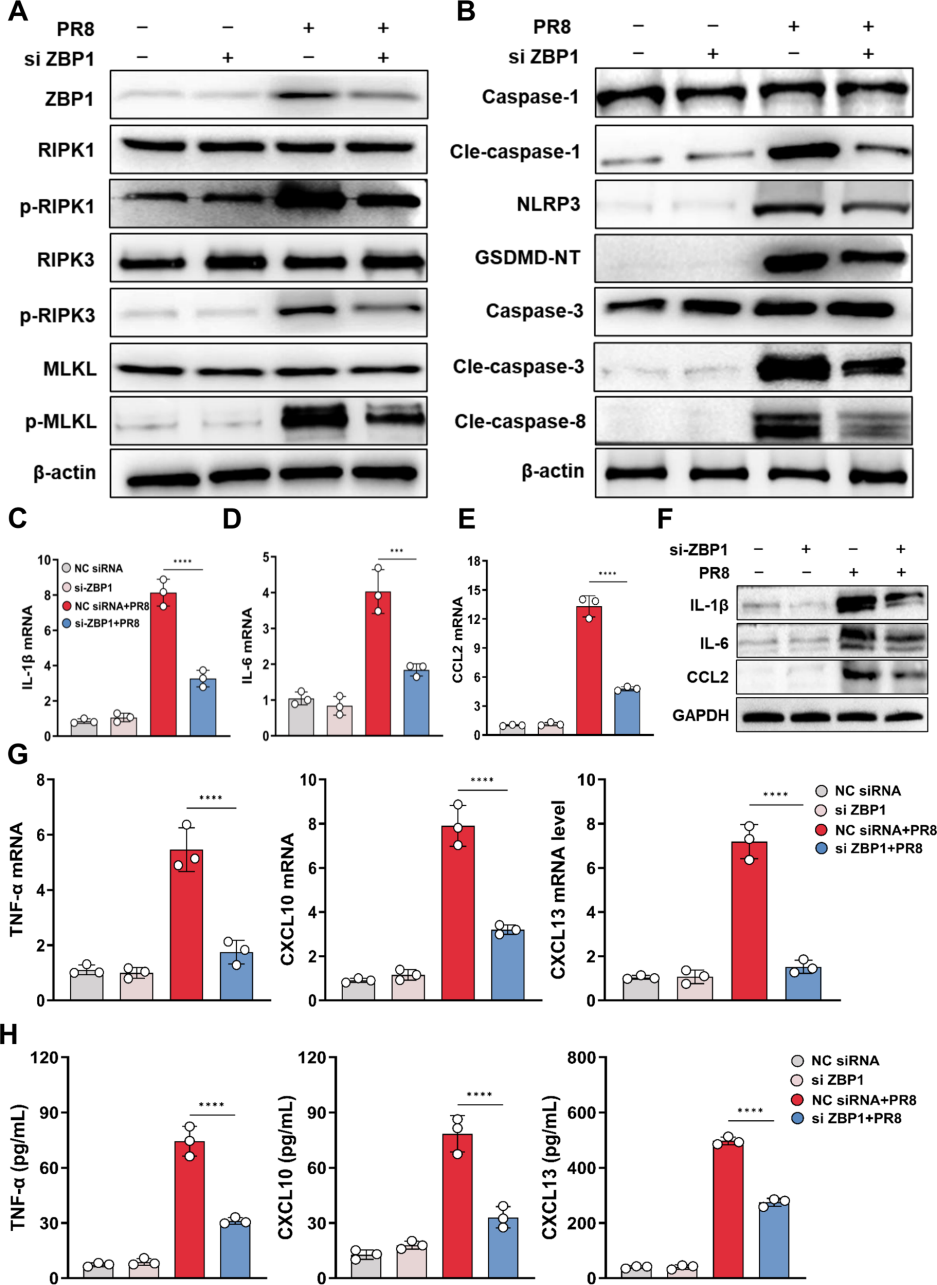
ZBP1 mediates IAV-induced PANoptosis and exacerbates the release of inflammatory chemokines. **A** A549 cells were transfected with 100 pmol ZBP1 siRNA for 48 h, and infected with PR8 for 12 h. Protein expression levels of ZBP1, RIPK1, p-RIPK1, RIPK3, p-RIPK3, MLKL, p-MLKL were detected by western blot. **B** A549 cells were transfected with 100 pmol ZBP1 siRNA for 24 h, and infected with PR8 for 12 h. Protein expression levels of caspase-1, cle-caspase-1, GSDMD-NT, NLRP3, caspase-3, cle-caspase-3, and cle-caspase-8 were detected by western blot. A549 cells were transfected with 100 pmol ZBP1 siRNA for 24 h, and infected with PR8 for 12 h. The transcription level of IL-1β (**C**), IL-6 (**D**), CCL2 (**E**) was detected by qRT-PCR (*n* = 3). (**F**) Protein expression levels of IL-1β, IL-6, and CCL2 were detected by western blot. **G** Transcription levels of TNF-α, CXCL10, and CXCL13 were detected by qRT-PCR (*n* = 3). **H** Secretion levels of TNF-α, CXCL10, and CXCL13 in cell supernatant (*n* = 3). Data are shown as mean ± SD compared to the NC siRNA + PR8 group. ***p < 0.001, ****p < 0.0001.

Given the elevated ZBP1 levels after viral infection, we hypothesized that ZBP1 mediates PANoptosis during IAV-induced inflammation. To test this, ZBP1 was silenced using siRNA in A549 cells prior to PR8 infection (MOI = 0.5, 12 h). Silencing ZBP1 reduced the expression of pyroptosis markers (GSDMD-NT cleaved caspase-1), apoptosis marker (cleaved caspase-3), and necroptosis marker (phosphorylated MLKL), as well as phosphorylated RIPK1 and RIPK3 (Fig. 4A, B). ZBP1 knockdown also decreased expression of cleaved caspase-8, further confirming its regulatory role in cell death. Additionally, interference with ZBP1 significantly downregulated mRNA levels of IL-1β, IL-6, and CCL2 (Fig. 4C–E). Protein levels of pro-inflammatory cytokines were also significantly reduced in interference with ZBP1 (Fig. 4F). Compared with control, interference with ZBP1 significantly reduced the mRNA levels of chemokines CCL2, CXCL10, and CXCL13 (Fig. 4G), and the protein levels of chemokines were also significantly reduced in BALF (Fig. 4H). These collective findings support that ZBP1 is a vital mediator of IAV-induced PANoptosis and downstream production of inflammatory cytokine and chemokine.

### GPR84 is activated in influenza virus-induced inflammation

**Fig. 5:**
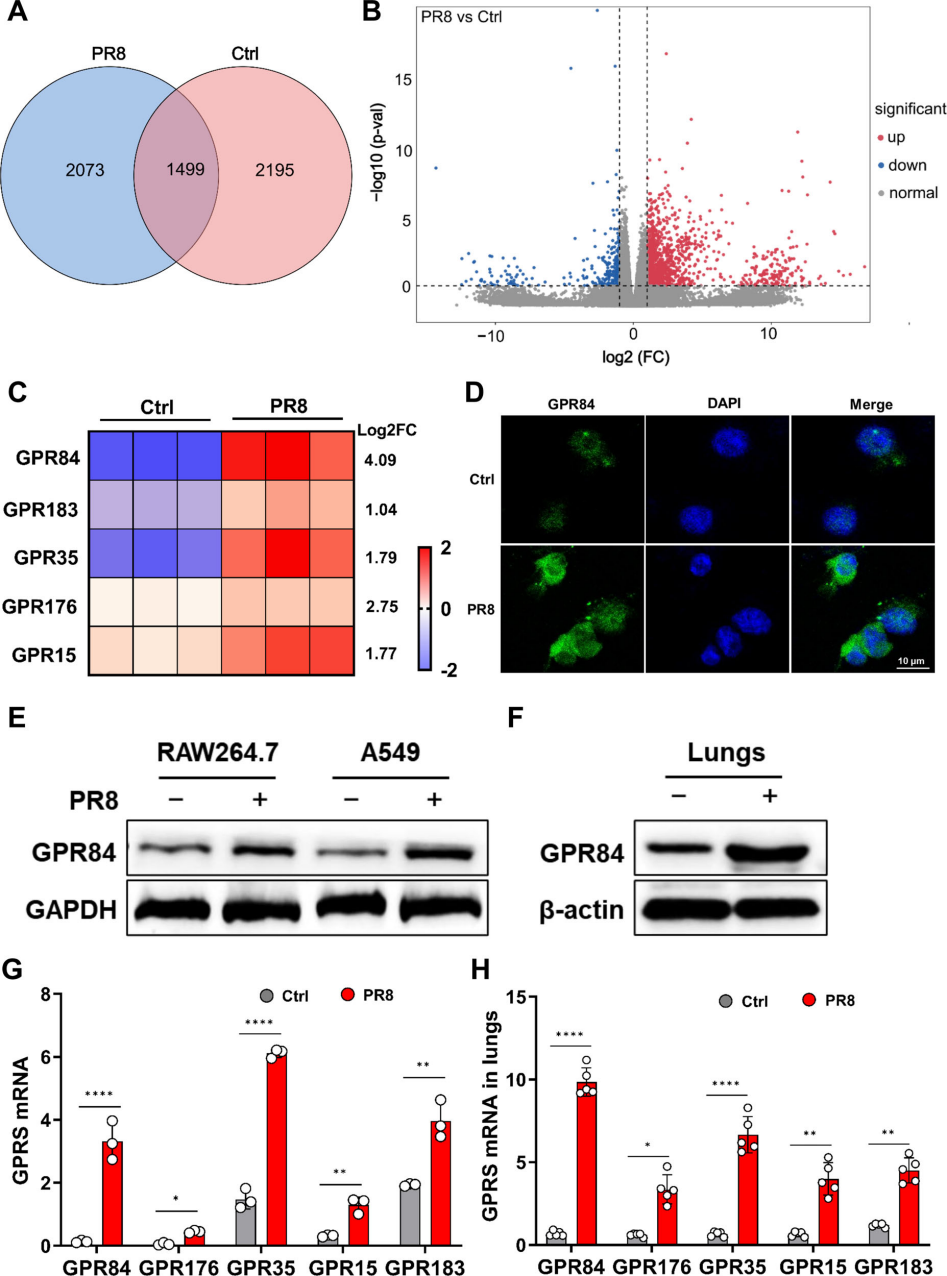
GPR84 is overexpressed in influenza virus-induced inflammation. **A** Venn diagram identifying 1499 differential genes across the model (PR8-infected) and blank (Ctrl) groups. **B** Volcano plot illustrating differentially expressed proteins (DEPs) between lung tissues of PR8-infected and blank groups. Points with ∣log2 (FC)∣ > 1, *p* < 0.05 are designated as DEPs. Red dots. represent up-regulated genes while blue ones represent down-regulated genes. **C** Heatmap depicting the levels of GPCRs between the PR8-infected group and the control group in the lungs of mice. **D** Immunofluorescence images of the GPR84 (green) in RAW 264.7 cells infected with PR8 for 12 h. Nuclei were counterstained with Hoechst 33342 (blue). Scale bar = 10 μm. **E** Western blot analysis of GPR84 protein level in RAW 264.7 and A549 cells. **F** Western blot analysis of GPR84 protein level in lung tissues. **G** Transcriptional levels of GPR84, GPR176, GPR35, GPR15, and GPR183 in A549 cells were measured by qRT-PCR. **H** mRNA expression levels of GPR84, GPR176, GPR35, GPR15 and GPR183 were detected by qRT-PCR in lung tissues. The above data shows an average ± SD. *p < 0.05, **p < 0 .01, ****p < 0.0001 compared to the control group.

To explore the mechanisms underlying IAV-induced inflammation, transcriptomic sequencing of lung tissues was conducted, focusing on G protein-coupled receptors (GPCRs). Differential expression analysis identified 1499 DEGs, 1215 upregulated and 284 downregulated in the infected group (Fig. 5A, B). Heatmap analysis highlighted significant upregulation of GPCRs including GPR15, GPR35, GPR84, GPR176, and GPR183, with GPR84 showing the most notable increase-approximately 4-fold (log2FC) compared to controls (Fig. 5C). Immunofluorescence analysis using fluorescently labeled anti-GPR84 antibodies further supported enhanced GPR84 protein expression in RAW264.7 cells (Fig. 5D). Consistently, GPR84 protein levels were markedly elevated in A549 cells, as well as in lung tissues from infection models (Fig. 5E, F). In parallel, qRT-PCR analysis validated the upregulation of GPR84 mRNA in H1N1-infected A549 cells and lung tissues (Fig. 5G, H). Together, these data suggest a potential role of GPR84 in modulating IAV-associated inflammation. To investigate the potential relationship between GPR84 and the downstream ZBP1-PANoptosome, we performed molecular docking analysis to simulate the binding pattern of GPR84 and ZBP1. Structural prediction using AlphaFold Server implicated that GPR84 and ZBP1 can form stable complex, with a calculated binding free energy of −13.9 kcal/mol. Specifically, ZBP1 residues SER-169, THR-180, and ARG-172 were predicted to form hydrogen bonds or electrostatic interactions with GPR84 residues SER-10, ASN-8, and ARG-174 (Fig. S3A). To further validate this interaction experimentally, we performed a cellular thermal shift assay (CETSA), which demonstrated that GPR84 enhanced the thermal stability of ZBP1 in a temperature-dependent manner (Fig. S3B). Moreover, drug affinity responsive target stability (DARTS) assays revealed that GPR84 increased ZBP1 resistance to protease digestion in vitro (Fig. S3C). Collectively, these findings support a model in which GPR84 interacts with and stabilizes ZBP1, thereby potentially facilitating downstream ZBP1–PANoptosome signaling and amplifying IAV-induced inflammatory responses.

### GPR84 knockout attenuates influenza virus-induced PANoptosis

**Fig. 6:**
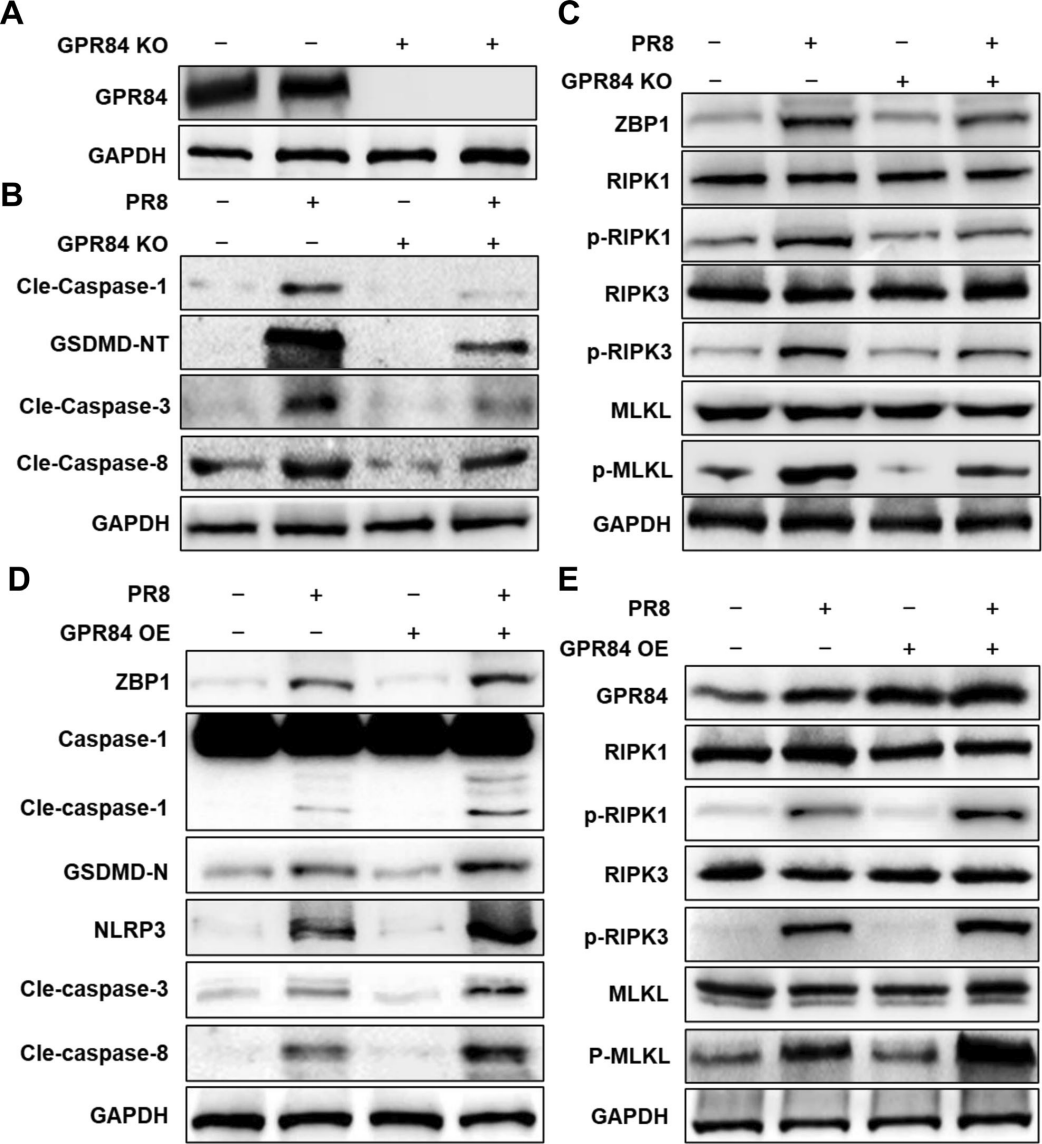
GPR84 deletion attenuates influenza virus-induced PANoptosis. Effect of knockout or overexpression of GPR84 on IAV-induced PANoptosis. A549 cells were transduced with lentivirus containing GPR84 sgRNA, or GPR84 overexpression plasmids for 48 h and infected with PR8 for 12 h. **A** Immunoblotting of the GPR84 protein level in A549 cells. **B** Protein expression levels of cle-caspase-1, GSDMD-NT, NLRP3, cle-caspase-3, and cle-caspase-8 in A549 cell lysates were analyzed by western blot. **C** Protein expression levels of ZBP1, RIPK1, p-RIPK1, RIPK3, p-RIPK3, MLKL, p-MLKL in A549 cell lysates were analyzed by western blot. **D** Protein expression levels of ZBP1, caspase-1, cle-caspase-1, GSDMD-NT, NLRP3, cle-caspase-3, and cle-caspase-8 were assessed by western blot in GPR84 overexpressing cells. **E** Western blotting analysis of GPR84, RIPK1, p-RIPK1, RIPK3, p-RIPK3, MLKL, p-MLKL levels in A549 cells.

**Fig. 7:**
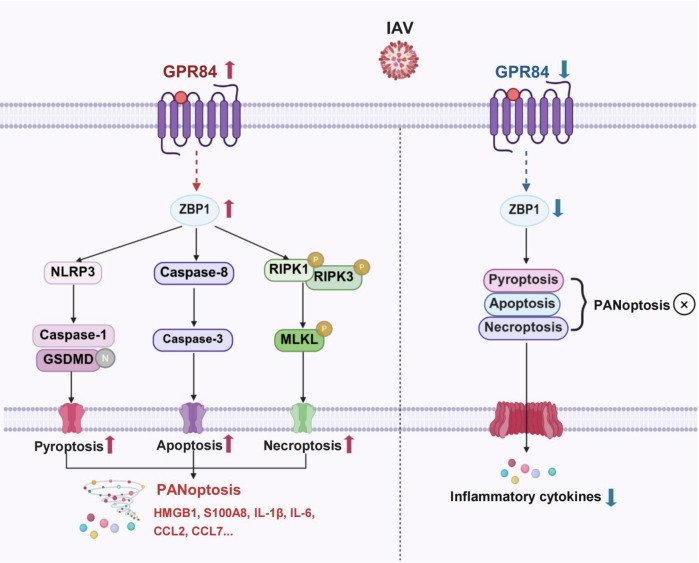
Proposed model of how GPR84 promotes influenza A virus-induced PANoptosis. GPR84 plays a central role in IAV-induced inflammation by promoting PANoptosis and the release of inflammatory mediators. Inhibition of GPR84 reduces ZBP1-mediated apoptosis and alleviates IAV-induced lung inflammation.

To examine the role of GPR84 in PANoptosis, GPR84-knockout A549 cells were generated using the LentiCRISPR V2 system (Fig. 6A). Upon IAV infection, wild-type cells showed elevated expression of PANoptosis markers, whereas GPR84-deficient cells displayed reduced levels of GSDMD-NT, NLRP3, cleaved caspase-1, cleaved caspase-3, and cleaved caspase-8 (Fig. 6B). GPR84 knockout also diminished ZBP1 expression and phosphorylation of RIPK1, RIPK3, and MLKL (Fig. 6C). Conversely, overexpression of GPR84 in A549 cells upregulated markers of pyroptosis (NLRP3, cleaved caspase-1, GSDMD-NT), apoptosis (cleaved caspase-3, cleaved caspase-8), and necroptosis (p-MLKL, p-RIPK1, and p-RIPK3) (Fig. 6D, E). Moreover, mice were administered GLPG1205 (30 mg/kg/day) for three consecutive days prior to PR8 infection. Lung tissues and BALF were collected on day 5 post infection for analysis. Histological examination revealed that GLPG1205 markedly attenuated inflammatory cell infiltration into the lung parenchyma and reduced pulmonary hemorrhage (Fig. S4A), accompanied by a decreased lung index (Fig. S4B). At the molecular level, GLPG1205 treatment significantly reduced the mRNA expression of pro-inflammatory cytokines, including IL-1β, IL-6, and TNF-α, in lung tissues at a dose of 30 mg/kg (Fig. S4C). Consistently, protein levels of these cytokines in BALF were also significantly decreased following GLPG1205 administration (Fig. S4D). Immunofluorescence staining of lung sections demonstrated that a significant reduction in GPR84-positive cells in the GLPG1205-treated group compared with the control (Fig. S4E), indicating that GLPG1205 acts as an antagonist on GPR84 in vivo. Furthermore, Western blot analysis showed that GLPG1205 suppressed IAV-induced upregulation of PANoptosis-associated proteins (Fig. S5), consistent with the phenotype observed in GPR84 deficient cells. Collectively, these results indicate that pharmacological inhibition of GPR84 mitigates IAV-induced lung injury and inflammatory responses. These results suggest that GPR84 is a critical regulator of IAV-induced PANoptosis and plays a significant role in viral pneumonia pathogenesis (Fig. 7).

## Discussion

IAV is a common and highly contagious respiratory virus, with IVP being a leading cause of death [33]. PANoptosis, a form of regulated cell death (RCD), involves the complex interactions and coordination among activation of pyroptosis, apoptosis, and necroptosis pathways [34]. This process plays a key role in various inflammatory pathological processes. Previous studies suggest that during IAV infection, ZBP1 activates the NLRP3 inflammasome through the RIPK1–RIPK3–Caspase-8 signaling axis, inducing pyroptosis, apoptosis, and necroptosis [35]. ZBP1 functions as a nucleic acid sensor, recognizing viral Z-RNA or Z-DNA via its Zα and RHIM domains [36]. In addition to IAV, such as VSV, HSV-1, and SARS-CoV-2 have been shown to trigger similar responses by upregulating ZBP1 expression in macrophages [36–39]. ZBP1 promotes the formation of a multiprotein PANoptosome complex, comprising inflammasome components (NLRP3, GSDMD, and caspase-1), apoptotic proteins (caspase-3, caspase-8), as well as necroptotic elements (RIPK3), thereby facilitating PANoptosis by activating the corresponding executors. However, excessive PANoptosis has been associated with pathological inflammation [40]. For instance, cytokines such as tumor necrosis factor TNF-α and interferon IFN-γ can synergistically induce PANoptosis, leading to multi-organ damage during cytokine storms and potentially fatal outcomes in sepsis or SARS-CoV-2 infection [38]. S100A8/A9, released by neutrophils, plays a dual role in inflammation, inducing inflammatory angiogenesis at low concentrations [40, 41] while promoting endothelial cell apoptosis at high concentrations [42]. Notably, TAK1 inhibition in macrophages has also been shown to trigger PANoptosis upon LPS or TNF-α stimulation [6, 43]. In our study, ZBP1-mediated PANoptosis was clearly activated in lung tissues of IAV-infected mice. The severity of inflammatory damage correlated positively with PANoptosis activity. Silencing ZBP1 markedly reduced the expression of PANoptosis-related genes (e.g., GSDMD, caspase-3/8, MLKL) and alleviated inflammation, suggesting that PANoptosis is a viable therapeutic target for viral-induced inflammatory diseases.

In the context of IAV, the pulmonary environment becomes a nexus of competing and converging lethal signals. The complex architecture of RCD in the virally injured lung contributes to the development of a cytokine storm, reflecting the convergence of multiple programmed cell death (PCD) pathways during viral pneumonia. Beyond PANoptosis, additional RCD modalities, including ferroptosis and autophagy-associated cell death, have been implicated in the pathogenesis of influenza-induced cytokine storms.

Ferroptosis, a distinct form of RCD characterized by iron-dependent lipid peroxidation, adds a layer of metabolic complexity to IAV-induced lung injury [44, 45]. Suppression or depletion of glutathione peroxidase (GPX4), the central enzyme responsible for detoxifying lipid peroxides, promotes ferroptosis, while GPX4 upregulation reduces reactive oxygen species (ROS) accumulation and inhibits ferroptotic death. The oxidative burst generated in macrophages during PANoptosis can amplify lipid peroxidation, thereby lowering the threshold for ferroptosis in neighboring epithelial cells. Unlike apoptosis, ferroptosis is lytically and structurally destructive [46]. Peroxidation of pulmonary surfactant lipids compromises alveolar membrane integrity, contributing to alveolar collapse—a pathological feature frequently observed in severe viral pneumonia.

Crosstalk between autophagy and PANoptosis further complicates this regulatory network. Autophagy, classically regarded as a cytoprotective process that clears viral components and damaged organelles, can be subverted by viruses to facilitate replication [47]. For example, blockade of autophagic flux by viral proteins such as matrix protein 2 (M2) leads to accumulation of the autophagy adaptor p62/SQSTM1. Excess p62 can function as a scaffold for NLRP3 inflammasome assembly, thereby promoting pyroptosis. Under conditions of sustained viral stress, excessive or dysregulated autophagy may shift from a homeostatic recycling mechanism to a pro-death pathway, contributing to necroptotic or apoptotic signaling through degradation of essential survival factors. Mitochondrial dysfunction represents another shared mechanistic axis. Mitochondrial membrane depolarization and subsequent release of mitochondrial DNA (mtDNA) into the cytosol activate the cGAS–STING pathway, thereby amplifying inflammatory signaling and sensitizing cells to both PANoptosis and ferroptosis [43, 48–50].

Collectively, these findings underscore that severe viral pneumonia is driven not by a single death pathway but by an interconnected network of cytotoxic programs. Accordingly, effective therapeutic strategies may require multimodal cytoprotection. Selective inhibition of a single pathway, such as necroptosis, may inadvertently exacerbate compensatory cell death mechanisms, including ferroptosis or apoptosis. A more rational approach involves targeting nodal regulators positioned at the intersection of these pathways. GPR84 represents one such candidate. Modulation of GPR84 signaling may rebalance the pulmonary microenvironment from uncontrolled, lytic cell death—characterized by overlapping ferroptotic, necroptotic, and pyroptotic processes—toward controlled resolution of inflammation. Such an approach could facilitate viral clearance while minimizing collateral tissue damage and reducing progression to acute respiratory distress syndrome (ARDS).

GPR84, a member of the GPR family, is increasingly recognized as a pro-inflammatory mediator. Its expression positively correlates with inflammation severity in multiple inflammatory conditions. GPR84 is widely expressed across multiple tissues, including the brain, lungs, liver, adipose tissue, gastrointestinal tract, and skeletal muscle [51–53]. Unlike other FFARs, which exert anti-inflammatory effects [54]. GPR84 expression is elevated in immune cells under inflammatory conditions. Elevated GPR84 mRNA levels have been observed in peripheral blood from patients with COVID-19 and sepsis [55, 56]. In a mouse model of acute lung injury (ALI) induced by intratracheal injection of LPS, GPR84 increased significantly in both bronchoalveolar lavage fluid and lung tissues, peaking approximately 12 h after stimulation [57]. Flow cytometry further identified alveolar macrophages and infiltrating neutrophils as major sources of GPR84 upregulation [26, 58]. GPR84 deficiency or pharmacological blockade reduced lung inflammation, neutrophil infiltration, and ROS production. Additionally, GPR84 activation enhanced CD11 expression in macrophages and stimulated NADPH oxidase-mediated ROS generation. Pro-inflammatory stimuli, including TNF-α and IL-1β, upregulate GPR84 expression [59–63]. GPR84 activation further promotes downstream signaling via phosphorylation of Erk1/2, Akt, p65, and STAT3, driving cytokine production [58, 59]. Although some evidence suggests that GPR84 may exert antiviral effects by limiting inflammatory responses [27–30], its specific role in IAV has not been fully elucidated. In this study, we hypothesized that inhibiting GPR84 could reduce IAV-induced PANoptosis, thus alleviating lung inflammation and disease progression. Transcriptome data from IAV-infected mice revealed significant GPR84 upregulation in lung tissue. Functional experiments showed that GPR84 overexpression exacerbated IAV-induced PANoptosis, while CRISPR-mediated knockout of GPR84 in A549 cells attenuated ZBP1-mediated PANoptosis, reducing expression of key mediators like GSDMD-NT, caspase-1, caspase-3, and caspase-8.

The clinical implication of PANoptosis and its regulation in our results may help explain why single-pathway inhibitors (e.g., Caspase-1 inhibitors targeting pyroptosis) or individual cytokine antagonists frequently demonstrated limited efficacy in clinical settings. Once a cell is committed to PANoptosis, blocking a single axis (e.g., necroptosis via RIPK3 inhibition) may simply redirect the one death signal toward alternative pathways (e.g., Caspase-8 mediated apoptosis) [64, 65]. GPR84 inhibitors such as PBI-4050 and GLPG1205, have shown therapeutic potential in inflammatory diseases [66]. Our findings suggest that, in the context of respiratory IAV infection, GPR84 contributes to inflammatory activation in the lung and that its inhibition effectively attenuates inflammatory responses. Specifically, pharmacological targeting of GPR84 with GLPG1205 suppressed PANoptosis-associated signaling and reduced the release of inflammatory mediators. Our findings advance the concept that effective suppression of PANoptosis and its downstream inflammatory cascade may be achieved by targeting upstream sensors and initiators, such as GPR84, rather than individual downstream effector pathways. By overcoming the intrinsic redundancy embedded within PANoptotic signaling, such strategies may offer a more clinically effective approach for the treatment of severe influenza and related viral pneumonias.

## Conclusion

Our findings demonstrate that GPR84 plays a central role in IAV-induced inflammation by promoting PANoptosis and the release of inflammatory mediators. Pharmacological inhibition of GPR84 attenuates ZBP1-mediated PANoptosis, reduces pulmonary inflammation, and improves disease outcomes in IAV-infected models. These findings highlight GPR84 as a promising therapeutic target for viral pneumonia and potentially other inflammation-driven diseases.

## Materials and methods

### Cells and reagents

RAW 264.7 cells were cultured in Dulbecco’s modified Eagle medium (DMEM). Human lung epithelial cells (A549) were kept in Ham’s F-12K medium (Procell, China). All medium were supplemented with 10% fetal bovine serum (FBS) and 1% penicillin-streptomycin. Cells were incubated at 37 °C in a humidified 5% CO_2_ incubator. The H1N1 strain A/PR/8/34 (PR8) was propagated in the allantoic cavities of 9-day-old pathogen-free chicken eggs. Inflammatory cytokines in samples collected from mouse blood or BALF were detected using ELISA kits (Adsbio). GLPG1205 was purchased

from TargetMol (T11411, Shanghai, China). Hoechst 33342 was purchased from Beyotime (Beijing, China). TRIzol reagent was supplied by Accurate Biology (Changsha, China). Fixative solution (4% formaldehyde, methanol-free) was purchased from Biosharp Life Sciences (Hefei, China). Dimethyl sulfoxide (DMSO) and Triton X-100 were ordered from Solarbio Science & Technology (Beijing, China). Primers used in this study are listed in Table 1, antibodies used for immunoblotting and immunofluorescence are listed in Table 2.

**Table 1:** qRT-PCR primer sequences.

Gene	Species	Primers sequence (5′-3′)
CCL2	Mouse	F: CCCAATGAGTAGGCTGGAGA
		R: CCTTAGGGCAGATGCAGTTT
CCL7	Mouse	F: CCACATGCTGCTATGTCAAGA
		R: ACACCGACTACTGGTGATCCT
CXCL10	Mouse	F: GATGACGGGCCAGTGAGAAT
		R: CTCAACACGTGGGCAGGATA
CXCL11	Mouse	F: ATGAACAGGAAGGTCACAGC
		R: GATGTCACATGTTTTGACGC
CXCL13	Mouse	F: ATTCAAGTTACGCCCCCTGG
		R: TTGGCACGAGGATTCACACA
HMGB1	Mouse	F: GCAGATGACAAGCAGCCCTA
		R: TTTTCAGCCTTGACCACCCC
S100A8	Mouse	F: AGGCCTTGAGCAACCTCATT
		R: AGTCATTCTTGTAGAGGGCATGG
S100A9	Mouse	F: CCTGGACACAAACCAGGACA
		R: GTGGGTTGTTCTCATGCAGC
TNF-α	Mouse	F: CATCTTCTCAAAATTCGAGTGACAA
		R: TGGGAGTAGACAAGGTACAACCC
IL-6	Mouse	F: GAGGATACCACTCCCAACAGACC
		R: AAGTGCATCATCGTTGTTCATACA
IL-1β	Mouse	F: GAAATGCCACCTTTTGACAGTG
		R: CTGGATGCTCTCATCAGGACA
GPR84	Mouse	F: CTCCTGCTACCATGAGTCTGT
		R: GTGCAGTAGAGTAGATCAGCCA
GPR183	Mouse	F: GTCGTGTTCATCCTGTGCTTCAC
		R: TCATCAGGCACACCGTGAAGTG
GPR35	Mouse	F: ACAAGGCAGGAACTGTG
		R: CCTAGGGCTCAGGCAGC
GPR176	Mouse	F: GGTGTTATGGTCAACTTGCCG
		R: AGAGCATCGTATAGATCCACCAG
GPR15	Mouse	F: CGTTATTATTGCGGTGGCGG
		R: TCTGGCTGGAACCCTGAAAC
GAPDH	Mouse	F: ACCCAGAAGACTGTGGATGG
		R: ACACATTGGGGGTAGGAACA
CCL2	Human	F: CATTGTGGCCAAGGAGATCTG
		R: CTTCGGAGTTTGGGTTTGCTT
CCL7	Human	F: ATGAAAGCCTCTGCAGCACT
		R: TCTGTAGCAGCAGGTAGTTGAAGT
CXCL10	Human	F: GTGGCATTCAAGGAGTACCTC
		R: TGATGGCCTTCGATTCTGGATT
CXCL11	Human	F: TTAAACAAACATGAGTGTGAAGGG
		R: CGTTGTCCTTTATTTTCTTTCAGG
CXCL13	Human	F: CGTGGGAATGGTTGTCCAAG
		R: TCCATTCAGCTTGAGGGTCC
TNF-α	Human	F: GAGGCCAAGCCCTGGTATG
		R: CGGGCCGATTGATCTCAGC
IL-6	Human	F: ACTCACCTCTTCAGAACGAATTG
		R: CCATCTTTGGAAGGTTCAGGTTG
IL-1β	Human	F: ATGATGGCTTATTACAGTGGCAA
		R: GTCGGAGATTCGTAGCTGGA
GAPDH	Human	F: GGGTGTGAACCATGAGAAGT
		R: GACTGTGGTCATGAGTCCT

**Table 2:** List of primary and secondary antibodies in this study.

Antibodies	Company	Cat#
NLRP3 Recombinant Rabbit Monoclonal Antibody [SC06-23]	Huabio	ET1610-93
Caspase-1 Recombinant Rabbit Monoclonal Antibody [SU40-07]	Huabio	ET1608-69
Gasdermin D (N terminal) Rabbit Monoclonal Antibody [PD00-18]	Huabio	HA721144
Caspase 3/p17/p19 Polyclonal Antibody	Proteintech	19677-1-AP
Caspase 8/p43/p18 Polyclonal Antibody	Proteintech	13423-1-AP
MLKL Recombinant Rabbit Monoclonal Antibody [SA40-04]	Huabio	ET1601-25
Anti-MLKL (phospho S358) Antibody [EPR9514]	Abcam	ab187091
RIP3 Rabbit Polyclonal Antibody	Huabio	ER1901-27
Phospho-RIP3 (S232) Recombinant Rabbit Monoclonal Antibody	Huabio	HA721428
Phospho-RIPK1 (Ser166) Polyclonal Antibody	Proteintech	28252-1-AP
RIPK1 Polyclonal Antibody	Proteintech	29932-1-AP
IGF2BP1(ZBP1) Polyclonal Antibody	Proteintech	22803-1-AP
GPR84 Rabbit Polyclonal Antibody	Abmart	PC15353S
HMGB1 Recombinant Rabbit Monoclonal Antibody [SA39-03]	Huabio	ET1601-2
IL-1 beta Recombinant Rabbit Monoclonal Antibody [PSH18-00]	Huabio	HA723965
CCL2 / MCP-1 Recombinant Rabbit Monoclonal Antibody	Huabio	HA723979
IL-6 Mouse Monoclonal Antibody [1–6]	Huabio	EM1701-45
S100A8 Recombinant Rabbit Monoclonal Antibody [PSH09-70]	Huabio	HA723135
S100A9 Recombinant Rabbit Monoclonal Antibody [JF096-8]	Huabio	ET1702-73
Bcl-2 Recombinant Rabbit Monoclonal Antibody [JF104-8]	Huabio	ET1702-53
Bax Recombinant Rabbit Monoclonal Antibody [SZ3-07]	Huabio	ET1603-34
FADD Recombinant Rabbit Monoclonal Antibody [PSH01-13]	Huabio	HA721625
ASC Recombinant Rabbit Monoclonal Antibody [PSH17-34]	Huabio	HA723917
AIM2 Rabbit Polyclonal Antibody	Huabio	HA500270
Beta Actin Monoclonal Antibody	Proteintech	60008-1-Ig
GAPDH Rabbit Polyclonal antibody	Elabscience	E-AB-40337
HRP-conjugated Goat Anti-Rabbit IgG (H + L)	Elabscience	E-AB-1003
HRP-conjugated Goat Anti-Mouse IgG (H + L)	Elabscience	E-AB-1001

### Animal studies

Animal experiments followed the Guidelines for the Care and Use of Medical Laboratory Animals and were approved by the Committee on the Ethics of Animal Experiments of Chengdu University of Traditional Chinese Medicine (Number: 2024007) and Fudan University (Number: 202309024S). Female BALB/c mice (6-8 weeks old) were obtained from laboratory animal technology (Beijing, China) and were kept in a specific pathogen free (SPF) facility. Mice were maintained on a 12-h light/dark cycle, at 24 °C ± 2 °C, and 50 ± 5% humidity, with unrestricted availability to nourishment and hydration. Mice were randomly assigned to control and model groups (*n* = 10 per group). The model group was intranasally inoculated with 50 μL of PR8 virus (10 LD_50_). After 5 days of infection, lung tissues from five mice per group were harvested to detect cytokine expressions.

### Histopathological staining and pathological evaluation

Lung tissues were dissected, fixed in 4% paraformaldehyde (PFA) for 72 h, dehydrated, and embedded in paraffin. Hematoxylin and eosin (H&E) staining was performed for histological analysis. On day 5 post-infection, mice were euthanized by cervical dislocation. Lungs were harvested and weighed to calculate lung index as an indicator of pulmonary edema and inflammation in the lungs. Lung index = lung wet weight (g)/body weight (g) × 100%.

### Immunofluorescence and fluorescent imaging

Lung sections were fixed in 4% paraformaldehyde for 15 min, permeabilized in 0.05% Triton X-100 for 15 min, and blocked with 3% BSA for 1 h. Primary antibodies were incubated overnight at 4 °C in blocking buffer. After washing, secondary antibodies were applied for 1 h at room temperature. Nuclei were counterstained with DAPI (1 μg/mL) for 15 min. Fluorescence microscopy was used for imaging analysis. For the cell-based model, A549 and RAW 264.7 cells infected with H1N1 were fixed, permeabilized, blocked, and incubated with appropriate primary and fluorescent secondary antibodies. Hoechst 33342 was used to visualize nuclear morphology. Imaging was performed and viewed using an Olympus FV2000 confocal microscope.

### Real-time quantitative polymerase chain reaction (qRT-PCR) analysis

Total RNA was extracted from the lung tissues of the mice and A549 cells with total RNA extractor (TRIzol) solution. RNA was quantified and transcribed using an Evo M-MLV RT kit (Accurate Biology, Changsha, China). The complementary DNA (cDNA) was diluted to perform qRT-PCR using the SYBR® Green Premix Pro Taq HS qRT-PCR Kit (Accurate Biology, Changsha, China) on a CFX96 Real-Time PCR Detection System (Bio-Rad, Hercules, CA, USA). GAPDH was used as an internal control. The relative expression level was calculated by the method of 2^−ΔΔCT^. Primer sequences are listed in Table 1.

### Immunoblotting

Proteins from cells or tissues were lysed, denatured, and separated by SDS-PAGE, followed by transfer to nitrocellulose membranes. Then, proteins were incubated in 1× Tris-buffered saline with Tween 20 (TBST) with 5% bovine serum albumin (BSA) for 1 h. With overnight incubation with primary antibodies at 4 °C, the membranes were sealed in the appropriate horseradish peroxidase (HRP)-conjugated secondary antibodies for 1 h. The protein bands on membranes were exposed using chemiluminescence kit (Abbkine, Wuhan, China) and imaged on the Tanon-1600 Imaging System (YPH-Bio, Beijing, China). The antibodies are listed in Table 2.

### Transcriptome data analysis

Left lung lobes were collected on day 5 post-infection from three biological replicates in each group for transcriptomic analysis. The libraries were sequenced on an Illumina Novaseq 6000 platform, and 150 bp paired-end reads were generated. Raw FASTQ files were processed using fastp for quality control. Clean reads were mapped to the reference genome using HISAT2, and gene expression levels were calculated. Read counts were obtained by HTSeq-count. Principal component analysis (PCA) was performed using R (v 3.2.0), and differentially expressed genes (DEGs) were identified with a threshold of *p* < 0.05 and ∣log₂ (fold change)∣ > 1.

### Small-interfering RNA transfection

All siRNAs were chemically synthesized by Hippobio (Huzhou, China). The siRNA sequences used were as follows: siZBP1 (F: 5′ -CCAGGUGAUUCCUCAACUUdTdT -3′, R: 5**’** -AAGUUGAGGAAUCACCUGGdTdG- 3′). A549 cells were seeded in 6-well plates and transfected with 100 nM siRNA per well via siRNA-Mate Plus (GenePharma, Shanghai, China). After 24–48 h, cells were harvested for RNA or protein analysis.

### Molecular docking

Molecular docking assays were performed using AlphaFold Server. The three-dimensional (3D) structures of GPR84 and ZBP1 were obtained from the PubChem database. The crystal structures of GPR84 (UniProtKB/Swiss-Prot: Q9NQS5) and ZBP1 (UniProtKB/Swiss-Prot: Q9H171) were prepared for docking simulations to predict potential binding interactions.

### Cellular thermal shift assay (CETSA)

A549 cells transfected with pCMV-GPR84-EGFP-3×FLAG-Neo plasmid or treated with DMSO culture were lysed using a liquid nitrogen freeze-thaw system. The soluble protein was transferred to PCR tubes and heated at 37, 41, 45, 49, 53, 57, and 61 °C for 3 min using a thermal cycler. After heating, samples were cooled and centrifuged at 12,000 × *g* for 30 min at 4 °C. The resulting supernatants were collected and analyzed by western blotting.

### Drug affinity responsive target stability (DARTS)

Proteins were collected from A549 cells transfected with GPR84 or treated with DMSO, washed with pre-chilled PBS, and lysed on ice for 30 min. Protein concentration was quantified and adjusted to 5 μg/μL. Samples were then digested with pronase for 30 min under the indicated conditions. Proteolyzed lysates were subsequently analyzed by western blotting.

### Generation of knockout cells by CRlSPR/Cas9 technology

GPR84 knockout A549 cells were generated by the LentiCRISPRv2 system, and GPR84 knockout A549 cells were generated accordingly. The specific oligonucleotides targeting the GPR84 gene were designed using the Cas9 target design tools (http://www.genome-engineering.org). The following oligoDNAs were used: GPR84-oligo DNA1, F: 5′ -CACCGatcgttatgttgcagttagc- 3′, R: 5′ -AAACgctaactgcaacataacgatC- 3′; GPR84-oligo DNA2, F: 5′ -CACCGctccacctgcactggcgcac- 3′, R: 5′ -AAACgtgcgccagtgcaggtggagC- 3’; GPR84-oligo DNA3, F: 5′ -CACCGagacagaattggaggcaaaa- 3′, R: 5′ -AAACttttgcctccaattctgtctC- 3′. The target guide sequence cloning protocol can be found at the Zhang Laboratory GeCKO Website (http://www.genome-engineering.org/gecko/).

### Statistical analysis

All the data are shown as the mean ± SD unless otherwise noted. Comparisons between two groups were made using unpaired *t*-tests. Statistical analyses were performed using GraphPad Prism 9. ImageJ was used for densitometric quantification of protein bands and fluorescence intensity. Comparisons between two groups were made using unpaired *t*-tests, while one-way ANOVA was used to analyze a dependent variable with more than two independent samples. A *p*-value < 0.05 was considered statistically significant between the experimental group and the control group. *P* values are represented as follows: *p < 0.05; **p < 0.01; ***p < 0.001; ****p < 0.0001; ns, not-significant values are not shown.

## Supplementary information

Supplementary figures and figure legends

Uncropped Western Blots

## Data Availability

Data and materials will be available upon reasonable request.
