# Supplementary material for: GPR84 aggravates lung inflammation through activating ZBP1-PANoptosome mediated PANoptosis following IAV infection
Source: Cell Death Discov. 2026 Jun 12;12:341. doi: 10.1038/s41420-026-03158-z (PMC13486761; doi:10.1038/s41420-026-03158-z)
Supplement: Supplementary file 1 [file 41420_2026_3158_MOESM1_ESM.docx]

**Supplementary figures and figure legends**

**Fig. S1: H1N1 PR8 infection induces PANoptosis-associated proteins *in vivo*.**

Six-week-old female BALB/c mice were inoculated with 10 LD_50_ PR8 virus intranasally. One group of mice administered PBS was mock-infected as a control, and the other group of mice were infected with PR8 virus.

**(A)** Protein expression levels of S100A8, S100A9, and HMGB1 were assessed in lung tissues by western blot.

**(B)** Protein expression levels of IL-1β, IL-6, and CCL2 in lung tissues were assessed by western blot.

**(C)** Levels of IL-1β, IL-6, and TNF-α secretion in serum, bronchoalveolar lavage fluid and lung homogenates (n = 5).

**(D)** Levels of CCL2, CXCL10, and CXCL13 secretion in serum, bronchoalveolar lavage fluid and lung homogenates (n = 5).

The above data show an average ± SD. * *p* < 0.05; ** *p* < 0.01; *** *p* < 0.001; **** *p* < 0.0001 compared to the control group.

**Fig. S2: H1N1 induces PANoptosis-related proteins by immunofluorescence.**

**(A-C)** Immunofluorescence images of the GSDMD-NT (red), cle-caspase-3 (red), and p-RIPK3 (red) in lung tissue at 5 dpi H1N1 infection. Nuclei were counterstained with Hoechst 33342 (blue). Scale bar = 150 μm.

**Fig. S3: Interaction between GPR84 and ZBP1.**

**(A)** Molecular docking showing the interaction between GPR84 and ZBP1 protein.

**(B)** CETSA was performed to analyze the thermal stability of ZBP1 across a temperature range of 37-61°C in A549 cells transfected with 2 μg of GPR84 plasmid for 48 h.

**(C)** DARTS was performed to measure the binding ability of GPR84 to ZBP1 in cell lysate.

**Fig. S4: GLPG1205 attenuates inflammation in H1N1 infected mouse model.**

Mice were orally administered GLPG1205 (30 mg/kg/day) once daily for three consecutive days prior to PR8 infection. Lung tissues were collected at 5 dpi for analysis.

**(A)** Representative histopathological images of lung sections at 5 dpi stained with H&E. Scale bar = 100 μm.

**(B)** Lung wet index was calculated as lung wet weight/body weight (n = 5).

**(C)** mRNA expression levels of IL-1β, IL-6, and TNF-α in lung homogenates at 5 dpi (n = 5).

**(D)** Protein levels of IL-1β, IL-6, and TNF-α secretion in BALF (n = 5).

**(E)** Immunofluorescence staining of GPR84 in lung tissues. Scale bar = 150 μm.

Data are presented as mean ± SD. **** *p* < 0.0001 compared to the PR8-infected group.

**Fig. S5: GLPG1205 suppresses PANoptosis signaling in H1N1-infected mice.**

Mice were orally administered GLPG1205 (30 mg/kg/day) once daily for three consecutive days prior to PR8 infection. Lung tissues were collected at 5 dpi for analysis.

**(A)** Protein expression of ZBP1, RIPK1, p-RIPK1, RIPK3, p-RIPK3, MLKL, p-MLKL were analyzed by western blot.

**(B)** Protein expression of AIM2, caspase-1, cle-caspase-1, NLRP3, GSDMD, GSDMD-NT, and ASC were analyzed by western blot.

**(C)** Protein expression of caspase-3, cle-caspase-3, caspase-8, cle-caspase-8, FADD, Bax, and Bcl-2 were assessed by western blot.
